# Thyroid carcinoma‐featured telomerase activation and telomere maintenance: Biology and translational/clinical significance

**DOI:** 10.1002/ctm2.1111

**Published:** 2022-11-17

**Authors:** Xiaotian Yuan, Huiyang Yuan, Ning Zhang, Tiantian Liu, Dawei Xu

**Affiliations:** ^1^ Laboratory Animal Center Shandong Provincial Hospital Affiliated to Shandong First Medical University Jinan China; ^2^ Department of Urology Qilu Hospital Cheeloo College of Medicine Shandong University Jinan China; ^3^ Department of Breast Surgery General Surgery, Qilu Hospital of Shandong University Jinan China; ^4^ Department of Pathology School of Basic Medical Sciences Cheeloo College of Medicine Shandong University Jinan China; ^5^ Department of Medicine Division of Hematology Bioclinicum and Center for Molecular Medicine (CMM) Karolinska Institutet and Karolinska University Hospital Solna Stockholm Sweden

**Keywords:** cancer biomarker, telomerase, TERT, TERT promoter mutations, thyroid carcinoma, thyroid nodule

## Abstract

**Background:**

Telomerase is a ribonucleoprotein complex consisting of a catalytic component telomerase reverse transcriptase (TERT), internal RNA template and other co‐factors, and its essential function is to synthesize telomeric DNA, repetitive TTAGGG sequences at the termini of linear chromosomes. Telomerase is silent in normal human follicular thyroid cells, primarily due to the *TERT* gene being tightly repressed. During the development and progression of thyroid carcinomas (TCs), *TERT* induction and telomerase activation is in general required to maintain telomere length, thereby conferring TC cells with immortal and aggressive phenotypes.

**Methods:**

The genomic alterations of the *TERT* loci including TERT promoter's gain‐of‐function mutations, copy number gain, fusion and rearrangements, have recently been identified in TCs as mechanisms to induce *TERT* expression and to activate telomerase. Importantly, numerous studies have consistently shown that TERT promoter mutations and *TERT* expression occur in all TC subtypes, and are robustly associated with TC malignancy, aggressiveness, treatment failure and poor outcomes. Therefore, the assessment of TERT promoter mutations and *TERT* expression is highly valuable in TC diagnostics, prognosis, treatment decision, and follow‐up design. In addition, the TERT promoter is frequently hypermethylated in TC cells and tumors, which is required to activate *TERT* transcription and telomerase. Dysregulation of other components in the telomerase complex similarly upregulate telomerase. Moreover, shortened telomeres lead to altered gene expression and metabolism, thereby actively promoting TC aggressiveness. Here we summarize recent findings in TCs to provide the landscape of TC‐featured telomere/telomerase biology and discuss underlying implications in TC precision medicine.

**Conclusion:**

Mechanistic insights into telomerase activation and TERT induction in TCs are important both biologically and clinically. The *TERT* gene aberration and expression‐based molecular classification of TCs is proposed, and for such a purpose, the standardization of the assay and evaluation system is required. Moreover, the TERT‐based system and 2022 WHO TC classification may be combined to improve TC care.

## Graphical abstract

Telomerase is activated in TCs, predominantly resulting from genomic, epigenetic and transcriptional aberrations in the *TERT* gene encoding the telomerase catalytic component. TERT promoter mutations and hypermethylation occur across TC subtypes and are associated with aggressive diseases. The assessment of telomerase biomarkers is highly valuable in TC diagnostics, prognostication and treatment decision. TERT/telomerase‐based TC molecular classification is suggested.

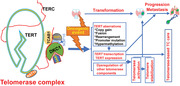

## 1 INTRODUCTION

Normal human somatic cells undergo a limited number of cell divisions and then enter a growth arrest or senescent state.^1^, ^2^, ^3^ In sharp contrast, malignant cells proliferate infinitely, which is a well‐recognized cancer hallmark. During carcinogenesis, genomic and epigenetic alterations cooperate to overcome the intrinsic senescence program to acquire this hallmark, among which telomeres and telomerase play an essential role.^2^ Telomeres are 6–20 kb TTAGGG repetitive sequences at the end of linear human chromosomes, and they, together with telomere‐binding proteins, maintain genomic stability and chromosomal integrity by inhibiting DNA damage response and illegitimate recombination.^1^, ^2^, ^3^ Telomeres are synthesized by telomerase, an RNA‐dependent DNA polymerase.^1^, ^2^, ^3^ In normal differentiated human cells, telomerase is generally silent, and progressive telomere attrition with their successive divisions occurs due to ‘the end‐replication problem’.^1^, ^2^, ^3^ Too short or dysfunctional telomeres activate a DNA damage response signalling, thereby inducing cellular senescence.^1^, ^2^, ^3^ Telomere shortening‐mediated senescence is thus a robust barrier to cellular immortality and transformation; and stabilizing telomere length is required for cancer cells to evade senescence for infinite proliferation.^2^, ^3^ It has been well established that activation of telomerase is the most common strategy by which cancer cells are empowered with an immortal phenotype.^2^, ^3^ Consistently, telomerase activity is detectable in up to 90% of human malignant tumours.^2^

Thyroid carcinomas (TCs) are the commonest malignancy in the endocrine system, and >95% of them are derived from follicular thyroid cells (thyrocytes), while the remaining (<5%) from calcitonin‐producing parafollicular or C cells.^4^, ^5^ Follicular cell‐originated TCs are primarily classified into papillary (PTCs, up to 85%), follicular (FTCs, 10%–15%), poorly differentiated (PDTCs) (5%–10%), and anaplastic (ATCs) subtypes (2%–3%).^5^, ^6^ Because PTCs and FTCs are usually differentiated tumours, they are together named differentiated TCs (DTCs).^5^ Like other malignancies, all these subtypes of TCs exhibit telomerase activation for their telomere maintenance, as revealed by numerous studies during last decades.^7^ Underlying mechanisms may be shared by different kinds of cancer but can also be highly cancer type‐specific.^2^, ^7^, ^8^ In a recent study, Montero‐Conde et al. dissected the telomerase‐based immortal phenotype in TCs comprehensively and they identified that both genomic and epigenetic aberrations, together with dysfunctional telomeres, contributed to telomerase activation in TCs, thereby promoting aggressive diseases.^9^ In the present review, we summarize recent advances to provide a fresh perspective on TC‐featured telomere/telomerase biology and translational/clinical implications.

## 2 TELOMERASE ACTIVATION BY MULTI‐MECHANISMS IN TCS

**FIGURE 1:**
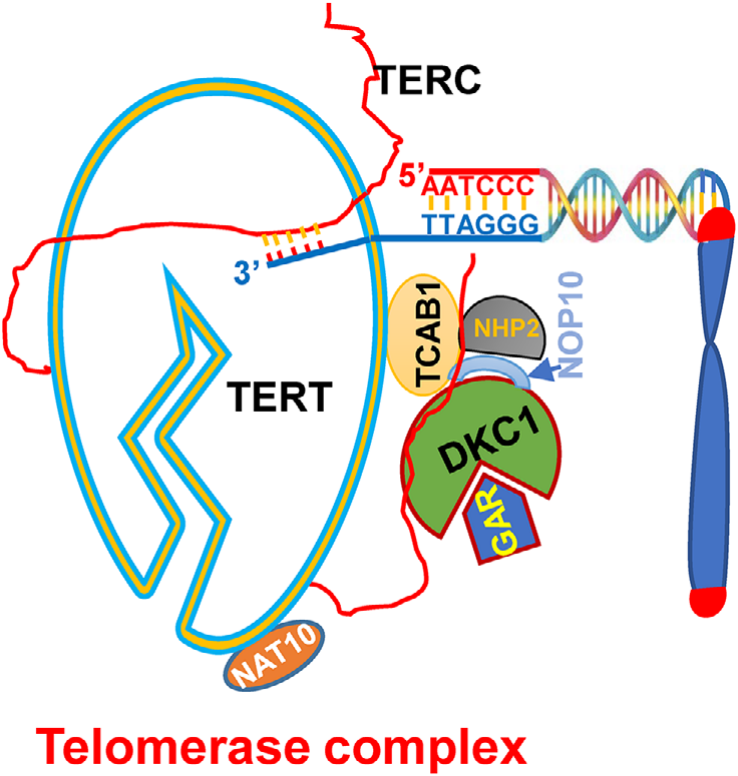
The schematics of the telomerase complex and telomerase‐mediated telomere‐lengthening. Telomerase as an RNA‐dependent DNA polymerase is composed of telomerase reverse transcriptase (TERT), non‐coding RNA template (TERC) and accessory proteins or co‐factors. TERT binds to TERC to form the telomerase core holoenzyme that synthesizes telomeric TTAGGG repeats using TERC‐containing CUAAC sequences as a template. TERT is also directly associated with *N*‐acetyltransferase number 10 (NAT10), and their interaction enhances enzymatic activity. TERC stably binds to the dyskerin complex consisting of dyskeratosis congenita 1 (DKC1), NOP10, NHP2 and GAR1; and the dyskerin complex is required for TERC stability. TERC also binds telomerase Cajal body protein 1 (TCAB1), the factor responsible for telomerase trafficking, assembling and function.

Human telomerase is a multi‐unit complex, and as an RNA‐dependent DNA polymerase, its core enzyme consists of telomerase reverse transcriptase (TERT), the component catalysing telomere extension and internal template‐containing telomerase RNA (TERC).^10^ TERC is ubiquitously expressed, whereas the *TERT* gene is tightly repressed in the vast majority of normal human somatic cells, which is a key event to silence telomerase in these cells.^2^, ^11^ Thus, the transcriptional activation of the *TERT* gene is required for transformed cells to acquire telomerase activity in the TC pathogenesis.^7^, ^11^ Given the essential role of TERT in telomerase activation for the molecular pathogenesis of human malignancies, great efforts have been made to define various impacts of TERT on development and progression of cancers, including TCs.^7^, ^11^ However, although TERT, together with TERC, is sufficient to reconstitute telomerase activity, such enzymatic activity is minimal.^10^ As described earlier, telomerase is a multi‐unit complex with a molecular weight of approximately 500 kDa (based on gel filtration), and several accessory proteins or co‐factors in the complex are required for enzymatic biogenesis and full functionality,^10^ whereas the aberrant expression and/or function of these co‐factors substantially affects telomerase activity. Dyskeratosis congenita 1 (DKC1) and its partners NHP2, NOP10 and GAR1, a pseudouridylation enzyme complex, and TCAB1 are stably associated with TERT and TERC and required for in vivo telomerase function (Figure 1).^10^ In addition, NAT10, *N*‐acetyltransferase‐like protein, is present in the telomerase complex by binding to TERT (Figure 1).^12^ The mutations or deficiency of these co‐factors lead to defective telomerase function and accelerated telomere erosion.^10^ Therefore, to thoroughly elucidate telomerase activation and functional relevance in TCs, it is necessary to examine these co‐factors in addition to TERT and TERC.

### 2.1 Genomic events inducing *TERT* transcription for telomerase activation in TCs

The recent mapping of cancer genomic landscapes has unravelled novel mechanisms to activate *TERT* transcription, which includes rearrangements or amplification of the *TERT* loci, and recurrent TERT promoter mutations.^2^ In TCs, all these genomic events are similarly observed and serve as major mechanisms to induce the *TERT* gene transcription for telomerase activation, which are our focus for discussion here.

#### 2.1.1 TERT promoter hotspot mutations

**FIGURE 2:**
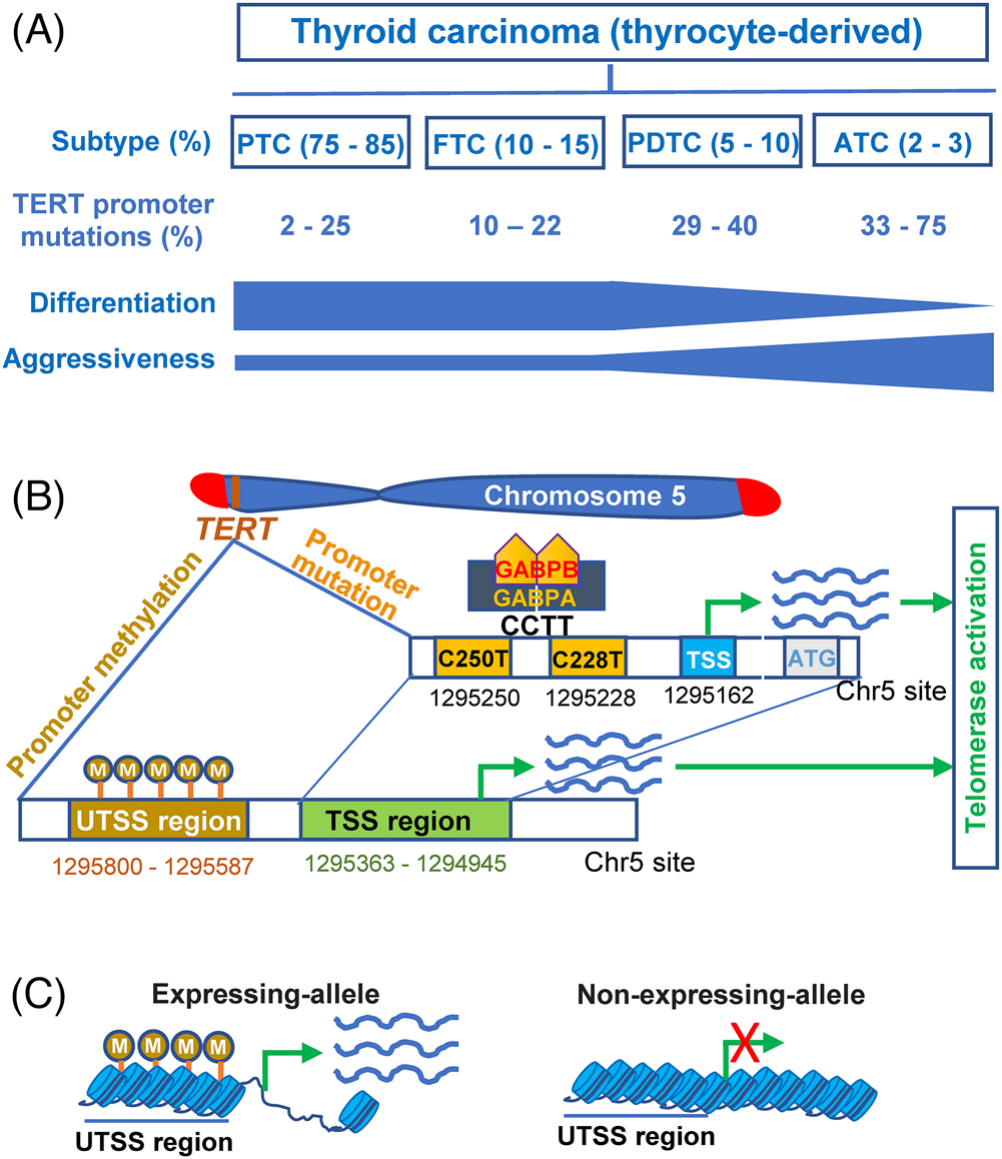
telomerase reverse transcriptase (TERT) promoter mutations and methylation for telomerase activation in thyroid carcinomas (TCs). (A) Frequencies of TERT promoter mutations (C228T and C250T) and association with cell differentiation and aggressiveness across four subtypes of TCs (see Refs. [11, 15] for details). PTC and FTC belong to differentiated thyroid carcinomas (DTCs), whereas poorly differentiated thyroid carcinoma (PDTC) and ATC are poorly differentiated and undifferentiated tumours, respectively. On the other hand, PDTC and ATC are the most aggressive TCs with the highest frequency of TERT promoter mutations. (B) The TERT promoter mutations (C228T or C250T), occurring in subsets of TCs, create a de novo ETS binding motif, and the GABPA‐GABPB1 complex binds this site, thereby activating *TERT* transcription and telomerase. On the other hand, oncogenic events result in the featured TC methylation pattern: Hypermethylation in the TERT promoter upstream of transcription starts site (UTSS, between 1295 800 and 1295 587 in Chr5), whereas hypomethylation in the transcription starts site (TSS, between 1295 363 and 1294 945) region. Of note, the TSS region is the proximal TERT promoter where two hotspot mutations take place. (C) TC and other malignant cells may exhibit monoallelic or biallelic *TERT* transcription. In cells with a monoallelic TERT expression, the expressing‐allele is characterized by the hypermethylated UTSS and hypomethylated TSS profile, which leads to open chromatin for *TERT* transcription. In contrast, the non‐expressing‐allele lacks this methylation pattern coupled with a closed chromatin.

Two hotspot mutations with a cytidine‐to‐thymidine (C > T) dipyrimidine transition occur at the proximal region of the TERT promoter (−124 and −146 bp from the ATG), and they are named C228T (or −124C > T) and C250T (or −146C > T), respectively. Across TC subtypes, the presence of C228T and C250T is mutually exclusive, whereas the C228T mutation is predominant (Figure 2A,B). Numerous published TC studies have revealed several featured properties. First, TERT promoter mutations are highly age‐dependent. In DTCs, including PTCs and FTCs, the mutation is rarely observed in young (<45 years) and paediatric patients.^11^, ^13^ Morton et al. analysed 359 radiation‐related and 81 sporadic PTC patients with mean age 28 years old (range 10.0–45.6) and only found the mutation in one patient (40.7 years old).^14^ Second, the presence of TERT promoter mutations is the featured hallmark for TC dedifferentiation and aggressiveness (Figure 2A). A recent meta‐analysis of 11 382 patients with DTC showed that average TERT promoter mutation rates were 10.6% (2%–25%) and 15.1% (10%–22%) for PTC and FTC, respectively,^15^ whereas PDTCs and ATCs had the mutation prevalence at 29%–40% and 33%–75%, respectively (Figure 2A).^13^, ^16^, ^17^, ^18^, ^19^, ^20^, ^21^ Montero‐Conde et al. showed a robustly higher frequency of TERT promoter mutations in aggressive TC tumours (45.7%) compared with those in disease‐free TCs (3.4%).^9^ Third, TERT promoter and BRAF or RAS mutations co‐occur at a high frequency in DTCs.^18^, ^21^

TERT promoter mutations (both C228T and C250T) create de novo ETS binding motifs that are bound by the ETS transcription factors, thereby activating *TERT* transcription (Figure 2B).^22^ The GABPA and GABPB1 complex were the first identified ETS members to activate mutant TERT promoters (Figure 2B).^22^ Consistently, in ATC‐ and PTC‐derived cell lines, inhibiting GABPA or GABPB1 expression indeed leads to substantial downregulation at levels of TERT mRNA and telomerase activity; however, the GABPA/GABPB1 knock‐down had similar inhibitory effects on *TERT* transcription in TC and other cancer cells harbouring a WT TERT promoter.^23^, ^24^, ^25^ Moreover, in primary TC tumours, there is a significantly inverse correlation between *GABPA* and *TERT* gene expression.^23^ Intriguingly, the presence of TERT promoter mutations represents a featured hallmark for aggressive TCs, as described earlier, whereas GABPA and GABPB1 expression is downregulated in those tumours.^23^, ^25^, ^26^ Several lines of evidence suggest that GABPA and GABPB1 serve as tumour suppressors to inhibit TC aggressiveness.^26^ More recently, a few other ETS transcription factors have been shown to stimulate mutant TERT promoter activity in TC cells.^27^ Further studies are required to define the exact role that GABP and other ETS members play in regulating mutated TERT promoters and telomerase activity in TCs. Regardless of regulatory mechanisms, the mutation‐carrying TC tumours express high levels of TERT mRNA and telomerase activity.^11^, ^23^, ^28^, ^29^ In PTCs, all tumours carrying the mutant TERT promoter expressed TERT mRNA, whereas TERT mRNA was only detectable in approximately 1/3 of WT‐promoter‐bearing tumours.^11^, ^23^ Similar results were also obtained from analyses of the TCGA pan‐cancers, including TCs.^8^

#### 2.1.2 TERT gene amplification, fusion and rearrangements in TCs

The *TERT* gene is located on chromosome 5p, and its gain or amplification is widespread in many cancers.^8^, ^23^ The TCGA pan‐cancer analysis showed that *TERT*‐amplified tumours expressed the highest levels of TERT mRNA and telomerase activity (a gene expression signature for telomerase activity estimation was applied), which suggests a critical role of the *TERT* amplification in telomerase activation during carcinogenesis.^8^ PTC tumours are in general diploid, and *TERT* copy number gain is at a very low frequency (.3%–4%).^30^ The analyses of the TCGA PTC cohort pinpointed increased *TERT* copy numbers in 25 of 495 tumours (5%).^6^ Paulsson et al. determined *TERT* copies in follicular thyroid tumours and observed *TERT* gains in 8% (6/77) of FTCs.^31^ Unexpectedly, increased *TERT* copies occurred in 21% (4/19) of follicular tumours of uncertain malignant potential.^31^ There are limited data available about *TERT* gene gains/amplification in PDTCs and ATCs. Panebianco et al. reported *TERT* copy gain in 2/4 of PDTC/ATC tumours,^30^ whereas another study showed that 5/9 (56%) of ATCs harboured the amplification of chromosome 5p.^32^ Using a FISH assay, Montero‐Conde et al. identified *TERT* copy gains in 5/6 of ATC tumours.^9^ These observations indicate a widespread *TERT* gene amplification in PDTCs and ATCs.

Yoo et al. identified one tumour with PDE8B‐TERT fusion and one with a *TERT* structural rearrangement in nine widely invasive FTCs.^33^ TERT mRNA levels were >50‐fold higher in these two tumours compared to the remaining 7 tumours.^33^ PDE8B, required for thyroid function, is highly expressed in the thyroid gland, whereas the rearrangement leads to super‐enhancer hijacking. Both events consequently activated robust *TERT* transcription.^33^ In addition, the re‐analysis of the TCGA cohort of PTCs identified MTMR12‐TERT fusion in one tumour.^33^ These findings indicate that both TERT promoter mutations and other genomic alterations contribute to telomerase activation in FTCs. *TERT* gene fusion and rearrangements seem occasional events in PTCs, PDTCs or ATCs.^6^, ^16^ However, the MTMR12‐TERT fusion was not reported in the original study.^6^ Thus, to exclude a possible underestimation, it may be worthy of re‐evaluating available TC genomic data to ascertain the frequency of structural *TERT* gene alterations in these tumours.

### 2.2 The TERT promoter methylation for telomerase activation in TCs

The TERT promoter is embedded in a CpG island and unmethylated in normal human cells, which allows repressor binding to block *TERT* transcription.^34^ The aberrant TERT promoter hypermethylation has been observed in cancer tissues and cells, including TCs, and the hypermethylation in the upstream of the transcription start site (UTSS) identified as an epigenetic mechanism to induce TERT expression by erasing repressor binding.^34^ In normal thyroid tissues, where the *TERT* gene is transcriptionally silent, there exist hypomethylated UTSS and TSS or proximal TERT promoter regions, whereas TERT‐expressing PTC and FTC cell lines exhibit hypomethylated proximal promoter but hypermethylated UTSS (Figure 2B).^35^, ^36^ Intriguingly, further studies reveal that TC and other tumour cells exhibit monoallelic or biallelic *TERT* transcription.^36^, ^37^ In monoallelic cells, hypermethylation of the UTSS region coupled with the hypomethylated proximal TERT promoter is the featured pattern in the expressing‐allele, whereas the non‐expressing‐allele can be hypermethylated or hypomethylated across the TERT promoter and gene body (Figure 2C).^37^ For cancer cells with TERT biallelic‐expression, both alleles display similar methylation patterns.^37^

It is evident from the findings earlier that hypermethylation of the TERT UTSS region is required to activate *TERT* transcription in cancer cells, which is further supported by the detailed analysis of TC cells bearing mutant TERT promoters.^36^ In TC cells with a heterogenous TERT promoter mutation, the de novo generated ETS motif at the mutant‐allele is bound by GABPA to activate *TERT* transcription, and this allele displays the expression‐allele methylation profile: hypermethylated UTSS and hypomethylated TSS.^36^ In contrast, the WT‐allele has the methylation pattern as seen in normal thyroid tissues. Moreover, the mutant‐allele is associated with histone H3K4 trimethylation (H3K4me3) that marks active transcription, whereas the repressive histone H3K27me3 occupies the WT‐allele.^36^

Avin et al. analysed the TERT promoter methylation in two FTC cell lines from the same patient, one from the lymph node metastasis (FTC‐133) and the other from distant metastasis (FTC‐238), more malignant.^35^ The UTSS methylation level was significantly higher in FTC‐238 cells than in FTC‐133 cells, and *TERT* expression was fourfold higher in FTC‐238 cells. These results suggest that the UTSS hypermethylation is associated with not only *TERT* expression but also aggressive behaviour of TCs. Indeed, the study by Montero‐Conde et al. showed that clinically aggressive TC tumours exhibited significantly higher levels of the UTSS region methylation in the *TERT* loci.^9^

### 2.3 TERC upregulation in aggressive TCs

TERC is a 451 nucleotide‐long lncRNA and serves as an RNA template during telomerase‐mediated telomere synthesis.^3^ Despite readily detectable TERC expression in normal cells, its upregulation occurs widely in TERT‐expressing tumours.^8^ Non‐thyroid malignancy analyses suggest that TERC overexpression facilitates cancer formation and progression^8^, ^38^; however, little is known about TERC expression, regulation and role in TCs. The study by Montero‐Conde et al. showed robustly enhanced TERC expression in aggressive TCs. Moreover, TERC RNA levels were independently associated with progression‐free survival in TC patients, indicating a driver‐effect on the TC pathogenesis.^9^

The dysregulation of *TERC* expression in oncogenesis may be attributable to different mechanisms, including aberrant alterations in transcriptional and epigenetic statuses, oncogenic signalling activation, gene amplification and among others, which depend on cancer types.^3^ It is currently unclear how TERC expression is controlled in TCs. Of note, the half‐life of TERC RNA is more than 2 weeks in TERT‐positive, non‐thyroid cancer cells,^39^ and consistently, TERC was observed to be more enriched in primary tumours across the TCGA pan‐cancers.^8^ More importantly, TERC RNA is stabilized by Dyskerin, encoded by *DKC1* gene.^10^ DKC1 is one of components in the telomerase complex, and its defect leads to dramatical decline in TERC RNA stability through which telomerase activity is diminished, and telomere homeostasis is impaired.^10^ Interestingly, TERC and DKC1 overexpressions were observed to co‐occur in aggressive TCs,^9^ which raises the possibility of DKC1‐mediated stabilization of TERC RNA in these tumours.

### 2.4 Dysregulation of telomerase co‐factors in TCs

In addition to TERT and TERC composed of the telomerase holoenzyme, the telomerase complex contains several co‐factors (Figure 1); however, little has been known about their alterations and roles in TCs. By examining expression of 26 telomere maintenance genes, Montero‐Conde et al. identified three significantly up‐regulated telomerase co‐factors in aggressive TCs: DKC1, TCAB1 and NAT10.^9^ As described earlier, DKC1 is required for the stability of TERC transcripts, and furthermore, it also enhances telomerase activity directly or through its broad effects on RNA metabolism.^40^ TCAB1 has been shown required for telomerase assembling, trafficking and enzyme activity.^10^ TCAB1‐KO HeLa and normal stem cells exhibited 80% reduction in telomerase activity.^41^ NAT10 exerts a dual effect on telomerase, binding to TERT to enhance telomerase activity on one hand^12^ and activating *TERT* transcription on the other.^42^ Thus, the dysregulation of telomerase co‐factors may serve as a compensatory mechanism to maintain sufficient telomerase activity required for TC pathogenesis. Conceivably, the upregulation of these telomerase co‐factors amplifies telomerase function or other driving‐effects in TCs.

## 3 TELOMERE SHORTENING, TELOMERE POSITION EFFECT AND SIGNALLING ALTERATIONS IN TCS

**FIGURE 3:**
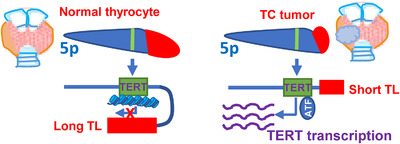
Telomere shortening‐mediated telomere position effect (TPE)/TPE over long distances (OLD) and metabolic alterations in thyroid carcinomas (TCs). Shorter telomere‐mediated TPE/TPE‐OLD. Long telomeres in normal thyrocytes form a telomere‐loop structure in the region near the telomerase reverse transcriptase (*TERT*) locus, resulting in a repressed TERT epigenetic state (left panel). Significant telomere shortening in TCs prevents the loop formation, thereby leading to opened chromatins, which allow the access to the TERT promoter by transcriptional activators and subsequent *TERT* transcription. ATF: activating transcription factor

During a stepwise carcinogenic process, significant telomere erosion readily occurs in precursor lesions before a full transformation is completed.^3^ Upon its activation in transformed cells, telomerase usually elongates the shortest telomeres to maintain them at a shorter length balance, because telomerase activity in most cancer cells is not high enough to restore all telomeres to the initial length.^3^ Therefore, shorter telomeres coupled with telomerase activation are paradoxically observed in most types of cancer. The role for telomerase/TERT in oncogenesis is overwhelmingly addressed, whereas the effect of shortened telomeres is explored insufficiently in TCs. The coexistence of shortened telomeres with *TERT* transcription and telomerase activation in the late stage of carcinogenesis has long been observed, but it remains elusive whether they have a causal relationship in TCs and other cancer types. As genes near telomeres and even at positions with a certain distance from telomeres can be regulated by telomere length, so‐called telomere position effect (TPE) and TPE‐over long distances (TPE‐OLD), shortened telomeres in premalignant and transformed cells may directly regulate gene expression to promote oncogenesis.^43^ The *TERT* gene has been shown to be controlled by TPE‐OLD (Figure 3).^43^ Long telomeres in normal cells form a telomere‐loop structure in the region near the *TERT* locus, resulting in a repressed TERT epigenetic state, whereas shortened telomeres in premalignant and cancerous cells fail to generate such a loop, thereby leading to opened chromatin, which allows the access to the TERT promoter by transcriptional activators (Figure 3).^2^, ^43^ Several TERT transcriptional activators, such as MYC and PAX8, are significantly upregulated,^2^, ^7^ and they and shorter telomeres act in concert to promote *TERT* transcription. Montero‐Conde et al. demonstrated that TPE‐OLD‐mediated TERT expression was indeed operative in TC tumours.^9^ They further showed that telomere shortening influenced chromatin configuration and gene expression widely, which are more robust in the 5 Mb ends of chromosomes 7p, 5p, 16p and 16q. The association between shortened telomeres and chromatin relaxation/gene expression significantly contributed to transcriptomic changes and aggressive behaviour of TCs.

Our unpublished analyses of the TCGA PTC cohort are largely consistent with the findings earlier. PTC tumours without telomere shortening exhibited significant enrichments of fat acid metabolism and oxidative phosphorylation pathways. Thus, PTC tumours without shortened telomere length maintain oxidative phosphorylation for their energy supply, indicating lack or low levels of a Warburg effect, and likely less malignancy. It is currently unclear how this happens. High TERT expression and its mitochondrial localization contribute to metabolic aberrations in (non‐thyroid) normal and cancer cells.^44^, ^45^ Further investigations are required to determine whether the links between telomere length and metabolism in PTCs are dependent on TPE/TPE‐OLD or other telomere length‐related activities in PTCs.

## 4 TELOMERASE ACTIVATION/TELOMERE MAINTENANCE IN TC PRECISION MEDICINE

### 4.1 TERT promoter mutations and mRNA expression as diagnostic markers for TCs

Thyroid nodules are extremely common in adults, and distinguishing between benign and cancerous ones is an essential precondition to avoid unnecessary treatment for patients with benign conditions and to provide appropriate cares for those with TCs. As TERT promoter mutations occur specifically in TCs, although it is absent in benign tumours and normal thyroid tissues, it should be a reliable TC biomarker. Thyroid samples from fine needle aspiration biopsy and core needle biopsy have been analysed for TERT promoter mutations.^46^, ^47^ Almost all results show satisfactory specificity for nodule diagnostics.

However, two issues have to be addressed here. First, discovering TERT promoter mutations in thyroid nodules that represent a malignant disease, however, in adult DTCs, including FTCs and PTCs, the prevalence of the TERT promoter mutation is <25% in Western countries while can be lowered to <3% in certain populations,^11^ which constitutes a relative low sensitivity for this biomarker. Thus, lack of TERT promoter mutations does not mean the exclusion of malignancy. The mutant TERT promoter combined with other genetic biomarkers is required to improve the accuracy of thyroid nodules diagnosis.^46^, ^48^ Second, highly sensitive, specific and simple assays are needed, and they should be suitable for clinical routine practise. Droplet digital PCR analyses have proven useful for assessing TERT promoter mutations in fine needle aspiration biopsy and core needle biopsy samples.^49^

The *TERT* gene is expressed in TC tumours with and without TERT promoter mutations, and theoretically, as a TC diagnostic marker improves sensitivity. However, TERT mRNA is detectable if the presence of lymphocytes or thyroiditis in benign nodules, which may compromise the specificity.^28^ Nevertheless, TERT mRNA assessment could be very helpful for thyroid nodule diagnosis if there are no substantial lymphocyte infiltrations.

### 4.2 TERT promoter mutations and gene expression as prognostic markers for TCs

Clinical investigations of TC patients have revealed the association of TERT promoter mutations with aggressive diseases, including advanced stages, extrathyroidal extension, vascular invasion, lymph node metastases, distant metastases and recurrence.^11^ Consistently, the direct evaluation of TERT promoter mutations and patient outcomes demonstrate its value as an independent factor in predicting shorter disease‐free survival in TC.^13^, ^17^, ^50^, ^51^, ^52^ In addition, most PTC tumours bear a BRAF^V600E^ mutation, and its coexistence with TERT promoter mutations leads to the formation of the most aggressive PTC tumours and even promotes the evolution of PTC to deadly ATC.^53^, ^54^, ^55^ Such a genetic combination could be applied to stratify the PTC patients with the highest mortality risk. A similar effect on PTC/FTC mortality was observed in the coexisting mutant RAS and TERT promoter mutations.^17^, ^51^, ^56^

The 2022 WHO classification of thyroid neoplasms categorizes follicular cell (thyrocyte)‐derived tumours into benign, low‐risk and malignant ones.^57^ The low‐risk tumours such as follicular tumours of uncertain malignant potential are morphologically and clinically intermediate between benign and malignant tumours and have the potential to develop metastasis, although the incidence is very low.^57^ Hysek et al. found TERT promoter mutations in 8 of 51 follicular tumours of uncertain malignant potential, and 3 of 8 eventually developed full‐blown malignant FTC, whereas none of the patients with a WT promoter underwent malignant progression.^58^ Thus, TERT promoter mutation analyses are useful for stratifying these patients with a transformation risk. In addition, we observed that the diagnostic hemithyroidectomy in one patient showed a featured FTA pathology but bearing the C228T mutation. The patient developed a metastatic FTC 2‐year post‐surgery.^59^ These findings suggest that the presence of TERT promoter mutations can help to identify thyroid nodules with malignant/aggressive potential even in the absence of malignant characteristics pathologically and morphologically.

Interestingly, Park et al. integrated the TERT promoter status into the WHO classification of FTCs, and they observed substantial improvements in outcome prediction.^60^ In all three subclasses of FTCs (minimally invasive‐, encapsulated angioinvasive‐ and widely invasive‐FTCs), the presence of TERT promoter mutations predicts significantly shorter disease‐free survival. Thus, the authors suggested a TERT promoter mutation‐based classification of FTCs.^60^ However, as described earlier, two out of nine widely invasive‐FTC tumours were identified to harbour *TERT* gene fusion or rearrangement, and both expressed dramatically high levels of TERT mRNA.^33^ In addition, increased *TERT* gene copies and hypermethylated promoters upregulate *TERT* expression, promoting FTC progression. Therefore, all these genomic and epigenetic alterations in *TERT* loci should be taken into consideration for the molecular classification of FTCs or other TC subtypes. In that setting, TERT mRNA assessment combined with TERT promoter analyses may be more appropriate. Indeed, higher *TERT* expression has been shown to predict shorter disease‐free survival in FTC and PTC patients.^23^, ^25^, ^28^, ^29^, ^31^ It will be interesting to determine whether the *TERT* gene expression‐based molecular classification of FTCs or PTCs outperforms that by the TERT promoter mutations.

### 4.3 Other telomerase/telomere‐related factors and TC care

In addition to TERT, Montero‐Conde et al. also observed TERC as an independent factor for progression‐free survival prediction of TCs.^9^ This finding may be especially important for TC tumours without detectable TERT expression. As many PTC tumours lack TERT expression or promoter mutations, it will be interesting to evaluate whether TERC can serve as a prognostic factor in those PTC patients. The authors further developed FISH‐based analysis of the chromosome 5p‐end region and telomere length for TC outcome prediction with promising results. The validation of these potential prognostic biomarkers in independent clinical studies is required.

## 5 CONCLUSIONS AND FUTURE PERSPECTIVES

**FIGURE 4:**
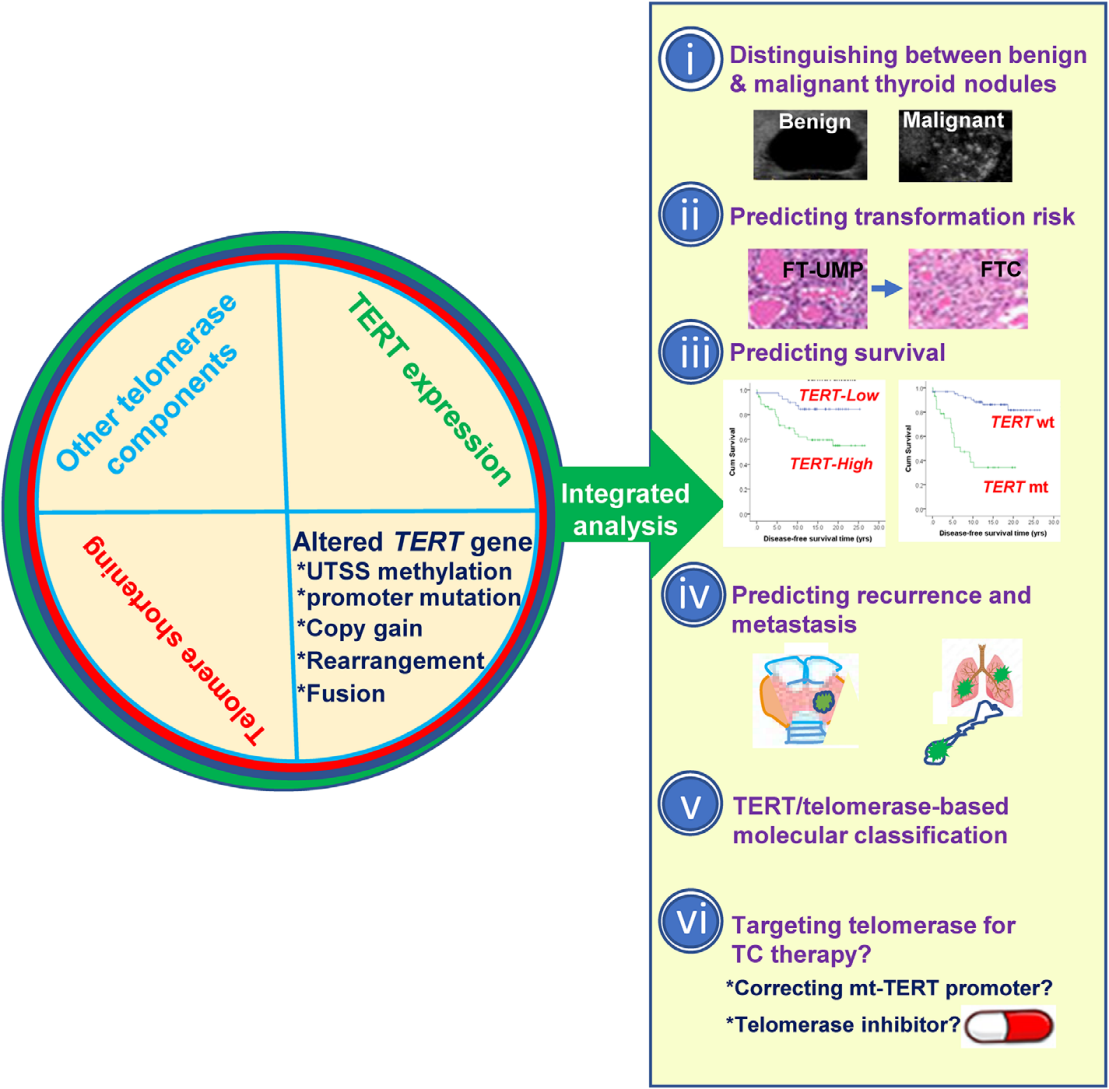
An integrated approach for telomerase‐based thyroid carcinoma (TC) precision medicine. Individual telomerase or telomere biomarkers, as shown in most clinical studies, have demonstrated their usefulness in TC precision medicine, including diagnostics and prediction of transformation risk, recurrence, metastasis and outcomes. It is conceivable that integrated analyses of all these telomerase/telomere‐related biomarkers may be more helpful. Moreover, the assay and evaluation system standardization of these biomarkers are required to establish a telomerase/telomerase reverse transcriptase (TERT)‐based molecular classification of TCs, which, together with the WHO classification, is expected to greatly improve TC care. In addition, the assay may also help identify TC patients suitable for telomerase/telomere‐based therapy, for instance, converting the mutated TERT promoter into a WT one in ATCs, and applying GRN163L to patients with TERT‐expressing PTCs and FTCs.

The studies of telomerase activation in TCs have not only led to a mechanistic understanding of TC‐featured telomerase biology and pathogenesis but also provided rationales for the development of telomerase‐based tools in TC care (Figure 4). For example, TERT promoter mutations and/or TERT expression have been shown to be useful biomarkers for TC diagnosis, risk stratification, prognosis, therapeutic decision‐making and follow‐up design. Park et al. evaluated the combination of TERT promoter mutation‐based molecular classification with the conventional WHO classification for FTC prognostication with improved sensitivity and accuracy.^60^ Because *TERT* expression is induced by not only TERT promoter mutations, but also other genomic and epigenetic (promoter methylation) aberrations, we would suggest *TERT* expression together with TERT promoter mutations rather than promoter mutations alone as the TERT‐based molecular classification of both FTCs and PTCs. Towards this purpose, the standardization of detection and evaluation systems is needed, and integrated analyses of telomerase/telomere‐related biomarkers may be more helpful (Figure 4). It should also be pointed out that telomerase components TERT and other co‐factors not only maintain telomere length but also exhibit oncogenic activities independently of telomere‐lengthening, further promoting TC development and progression.^2^ Therefore, it is rational to target telomerase for TC therapy. However, studies on telomerase‐based TC therapy have fallen behind. No clinical trials of telomerase inhibitors for TC treatment have been reported so far. Given a high frequency of TERT promoter mutations in deadly ATCs, targeting this genomic event is expected to hold great therapeutic promise and is worthy of investigative efforts.

## CONFLICT OF INTEREST

The authors declare that they have no conflict of interest.
